# Minimising inhaled corticosteroids for COPD

**DOI:** 10.4102/phcfm.v16i1.4756

**Published:** 2024-12-18

**Authors:** Benji Heran, Thomas L. Perry, Ken Bassett

**Affiliations:** 1 Department of Anaesthesiology, Pharmacology and Therapeutics, Faculty of Medicine, University of British Columbia, Vancouver, Canada

**Keywords:** inhaled steroids, corticosteroids, chronic obstructive pulmonary disease, chronic obstructive airways disease, primary care, therapeutics

## Abstract

This Therapeutic Letter considers the evidence for inhaled corticosteroids (ICS) as a treatment for Chronic Obstructive Pulmonary Disease (COPD). Drug therapy aims to alleviate symptoms, enhance functional capacity and prevent exacerbations, but has not consistently shown to reduce mortality or improve quality of life based on randomised trials.Inhaled corticosteroids have shown limited benefits for COPD symptoms and exacerbations but increased risks of serious harms. Guidelines recommend limiting ICS to severe COPD and only for repeated exacerbations. Studies show withdrawing ICS can be done safely for stable COPD patients with infrequent exacerbations, especially those with lower eosinophil counts. Provincial, national and international guidelines now recommend limiting ICS prescriptions to severe COPD stages. Long-term ICS use may lead to serious side effects, including pneumonia and fractures. Initial COPD therapy should focus on short-acting bronchodilators, not ICS. Adding long-acting bronchodilators is recommended before considering ICS because of limited benefits and risks of serious harms. For persistent symptoms, long-acting muscarinic antagonists (LAMA) or long-acting beta2 agonists (LABA) are recommended, with the addition of ICS reserved for those with repeated exacerbations and severe COPD. Deprescribing ICS can be considered in clinically stable patients, particularly for those with infrequent exacerbations and mild COPD. When applicable, tapering ICS over several months is advised for patients with elevated eosinophil counts. Overall, the risks of serious harms from ICS typically outweigh their limited benefits for mild COPD patients in primary care.

## Introduction

Chronic obstructive pulmonary disease (COPD) is characterised by airway inflammation and irreversible airflow obstruction that cause shortness of breath, cough and excess mucus production, reducing quality of life. Permanent anatomical changes make bronchodilators less effective for COPD than for asthma.

Cigarette smoking is the principal cause of COPD, although long-term exposure to other lung irritants (including air pollution) also contributes. Stopping smoking improves symptoms and is the only effective strategy to slow disease progression and reduce premature mortality.^1^

Inhaled corticosteroids (ICS) prescribed for patients with milder forms of COPD should be tapered down as they are not as efficacious as in patients with asthma and should rather be prescribed in patients with more severe COPD with repeated exacerbations. Drug therapy aims to alleviate symptoms, enhance functional capacity and prevent exacerbations, although clinical trials have not consistently shown a significant reduction in mortality in all stages of COPD severity or improved quality of life related to ICS.^2^

There is an urgent need to improve rational prescribing in primary health care (PHC) in the African region, to use limited resources more efficiently and to minimise adverse drug effects.^3,4,5,6,7^ One such way, is to consider deprescribing ICS in mild COPD (GOLD stages I and II), and clinically stable patients with infrequent exacerbations as the risks of serious harms, including pneumonia and fractures, typically outweigh their limited benefits for mild COPD patients. However, in patients with severe COPD (GOLD stage III and IV), ICS as part of triple therapy of more than 6 months duration, has been shown to reduce all-cause mortality risk especially in patients with eosinophil counts of > 200/UL.^8^

Clinical goals of drug therapy are to reduce symptoms, improve functional capacity and prevent exacerbations.

## Inhaled corticosteroids: The evidence

Recognising their efficacy for asthma, doctors began to prescribe ICS for COPD in the 1980s, without evidence from randomised controlled trials (RCTs).^9^ Two decades later, RCTs had shown no mortality benefit from ICS compared with placebo, no reduction in the proportion of people experiencing an exacerbation and no improvement in quality of life.^10,11,12^ A possible explanation is that while corticosteroids potently suppress eosinophilic airway inflammation in asthma, the neutrophilic inflammatory process in COPD is typically steroid resistant.^9^

Provincial, national and international guidelines all recommend limiting prescription of ICS to the most severe stages of COPD.^13,14,15^ During 2017–2021, 51 128 British Columbians initiated drug therapy for COPD, of whom 27% received an ICS alone or in combination. This proportion was stable over the 5 years.^16^ It is lower than in a large United Kingdom sample, in which 47% of COPD patients still received ICS as a component of initial daily drug therapy during 2015 (down from 77% in 2005).^17^

Serious harms from chronic ICS use include pneumonia and fractures,^18^ updated here from an unpublished 2020 Therapeutics Initiative meta-analysis (Table 1).^19^

**TABLE 1:** Risks of harm from inhaled corticosteroids use in chronic obstructive pulmonary disease.

Outcome (harm)	RCT’s reporting outcome	Comparison	Findings of meta-analysis
Pneumonia requiring hospitalisation	49 RCTs (*N* = 57 027) Fluticasone: 32 RCTs (*N* = 46 877) Budesonide: 17 RCTs (*N* = 10 150)	Fluticasone vs. non-ICS	RR 1.50 (95% CI: 1.34–1.68)†
			ARI 1.1%, NNH 93 (21 months)
		Budesonide vs. placebo	RR 1.60 (95% CI: 1.01–2.55)†
			ARI 0.5%, NNH 188 (9 months)
Total fractures	20 RCTs (*N* = 25 936) Fluticasone: 18 RCTs (*N* = 23 079) Budesonide: 2 RCTs (*N* = 2857)	Fluticasone or budesonide vs. non-ICS	RR 1.28 (95% CI: 1.07–1.54)
			ARI 0.42%, NNH 240 (16 months)

CI, confidence interval; RCT, randomised controlled trial; RR, relative risk; ARI, absolute risk reduction; NNH, number needed to harm; vs., versus.

† , independent of ICS dose, duration, or baseline severity of COPD.

## Chronic obstructive pulmonary disease guidelines discourage routine inhaled corticosteroids prescription

Rational drug therapy employs the simplest and least expensive treatment to achieve individual therapeutic goals. For *initial therapy*, Canadian (2020) and Global initiative for chronic Obstructive Lung Disease (GOLD, 2022) guidelines recommend short-acting beta agonists (SABA) or short-acting muscarinic antagonists (SAMA) to relieve shortness of breath.^13,15^ For *worsening symptoms or to reduce exacerbations*, both recommend short and then long-acting bronchodilators (long acting beta agonist [LABA] or long acting muscarinic antagonist [LAMA]), alone or in combination. To a combination of LAMA and LABA, add ICS at the lowest possible dose *only for people who continue to experience repeated exacerbations*. The GOLD initiative and a 2020 Cochrane systematic review point out that no ‘escalation’ strategy has been tested in RCTs,^15,20^ while de-prescribing ICS has been tested.

## Can inhaled corticosteroids be de-prescribed safely?

Two recent manufacturer-funded studies evaluated ICS withdrawal, focusing on acute exacerbations, the most relevant clinical issue. Among 2488 people with severe – very severe COPD who were susceptible to exacerbations, WISDOM (2014) *compared triple therapy* for up to 1 year (tiotropium 18 mcg/day + salmeterol 50 mcg twice/day + fluticasone 500 mcg twice/day) *with gradual withdrawal of fluticasone* over a 12-week period (followed by LAMA/LABA alone).^21^ *Moderate or severe exacerbations were similar among people who discontinued or continued ICS therapy* (hazard ratio, 1.06; 95% confidence interval [CI]: 0.94–1.19). There were no clinically important between-group differences in symptoms, quality of life or safety. No patient subgroup had increased likelihood of exacerbations after stopping ICS.

Among 1053 people *without frequent exacerbations*, SUNSET (2018) compared *continued triple therapy* for up to 26 weeks (tiotropium 18 mcg/day + salmeterol 50 mcg / fluticasone 500 mcg twice/day) *with abrupt discontinuation of ICS after long-term triple therapy*, replaced by once daily LAMA/LABA (indacaterol 110 mcg / glycopyrronium 50 mcg).^22^ Annualised *moderate or severe exacerbations did not differ* between treatments (rate ratio 1.08; 95% CI: 0.83–1.40). There was no difference in the time to first moderate or severe COPD exacerbation (hazard ratio 1.11; 95% CI: 0.85–1.46).

SUNSET is the only RCT to report a pre-specified analysis of patient subgroups defined by baseline blood eosinophil counts, one approach to differentiating COPD (<300 cells/µL) from asthma (≥ 300 cells/µL). When baseline eosinophil count was < 300/µL (COPD), stopping ICS made no difference to the rate of moderate or severe exacerbations, nor the time to exacerbation. In contrast, *in people with baseline eosinophils* ≥*300/µL (asthma/COPD overlap)*, stepdown to LAMA or LABA alone increased moderate or severe exacerbations versus continuing ICS over 6 months (rate ratio 1.86; 95% CI: 1.06–3.29).

## Conclusions

- Short-acting bronchodilators (SABA or SAMA), but not ICS, remain the initial drug therapy for COPD in primary care.
- For persistent symptoms or exacerbations, adding LAMA or LABA, then LAMA and LABA together, is recommended (but this strategy has not been tested in RCTs).
- For most people with COPD, the increased risk of serious harms from ICS outweighs the limited known benefits for symptoms or exacerbations.
- It is reasonable to try stopping ICS in clinically stable patients with infrequent exacerbations. For patients with eosinophils above 300/µL in a complete blood count, first taper ICS over a few months.
